# Cardiovascular magnetic resonance for therapy monitoring in cardiomyopathies

**DOI:** 10.1093/ehjimp/qyag153

**Published:** 2026-09-06

**Authors:** Manuel Garofalo, Giorgia Panichella, Alberto Clemente, Carmelo De Gori, Andrea Barison, Michela Chianca, Alberto Aimo, Giancarlo Todiere

**Affiliations:** Cardiomyopathy Unit, Careggi University Hospital, Florence, Italy; Cardiomyopathy Unit, Careggi University Hospital, Florence, Italy; Radiology Division, Fondazione Toscana Gabriele Monasterio, Pisa, Italy; Radiology Division, Fondazione Toscana Gabriele Monasterio, Pisa, Italy; Interdisciplinary Center for Health Sciences, Scuola Superiore Sant’Anna, Piazza Martiri della Libertà 33, Pisa 56127, Italy; Cardiology Division, Fondazione Toscana Gabriele Monasterio, via Moruzzi 1, 56124 Pisa, Italy; Cardiology Division, University Hospital of Pisa, via Roma 55, 56126 Pisa, Italy; Interdisciplinary Center for Health Sciences, Scuola Superiore Sant’Anna, Piazza Martiri della Libertà 33, Pisa 56127, Italy; Cardiology Division, Fondazione Toscana Gabriele Monasterio, via Moruzzi 1, 56124 Pisa, Italy; Cardiology Division, Fondazione Toscana Gabriele Monasterio, via Moruzzi 1, 56124 Pisa, Italy

**Keywords:** cardiomyopathies, cardiovascular magnetic resonance, late gadolinium enhancement, T1/T2 mapping, extracellular volume, treatment response monitoring

## Abstract

Cardiomyopathies are increasingly managed with disease-modifying and phenotype-specific therapies, creating a need for imaging biomarkers that can reliably capture treatment response over time. Cardiovascular magnetic resonance (CMR) is uniquely suited to this role because it combines highly reproducible assessment of cardiac structure and function with detailed tissue characterization. Cine imaging enables serial evaluation of ventricular volumes, mass, and ejection fraction; late gadolinium enhancement identifies focal replacement fibrosis and arrhythmogenic substrate; and native T1/T2 mapping with extracellular volume quantification provides quantitative markers of diffuse fibrosis, oedema, inflammation, and infiltrative burden. In this review, we discuss the role of CMR in therapy monitoring across the spectrum of cardiomyopathies. In dilated cardiomyopathy, CMR tracks reverse remodelling and helps identify persistent scar and non-response despite guideline-directed therapy or cardiac resynchronization. In non-dilated and arrhythmogenic cardiomyopathies, serial tissue characterization helps refine dynamic arrhythmic risk. In hypertrophic cardiomyopathy, CMR documents structural and interstitial changes during treatment with myosin inhibitors and after septal reduction. In amyloid and metabolic/storage cardiomyopathies, mapping techniques are particularly useful for demonstrating stabilization or regression of myocardial disease under targeted treatments. Overall, CMR offers a framework for standardized longitudinal follow-up and for the development of biologically meaningful surrogate endpoints in clinical trials. Wider harmonization of acquisition, analysis, and reporting will be essential to fully integrate CMR into individualized therapeutic decision-making.

## Graphical Abstract

**Graphical Abstract:**
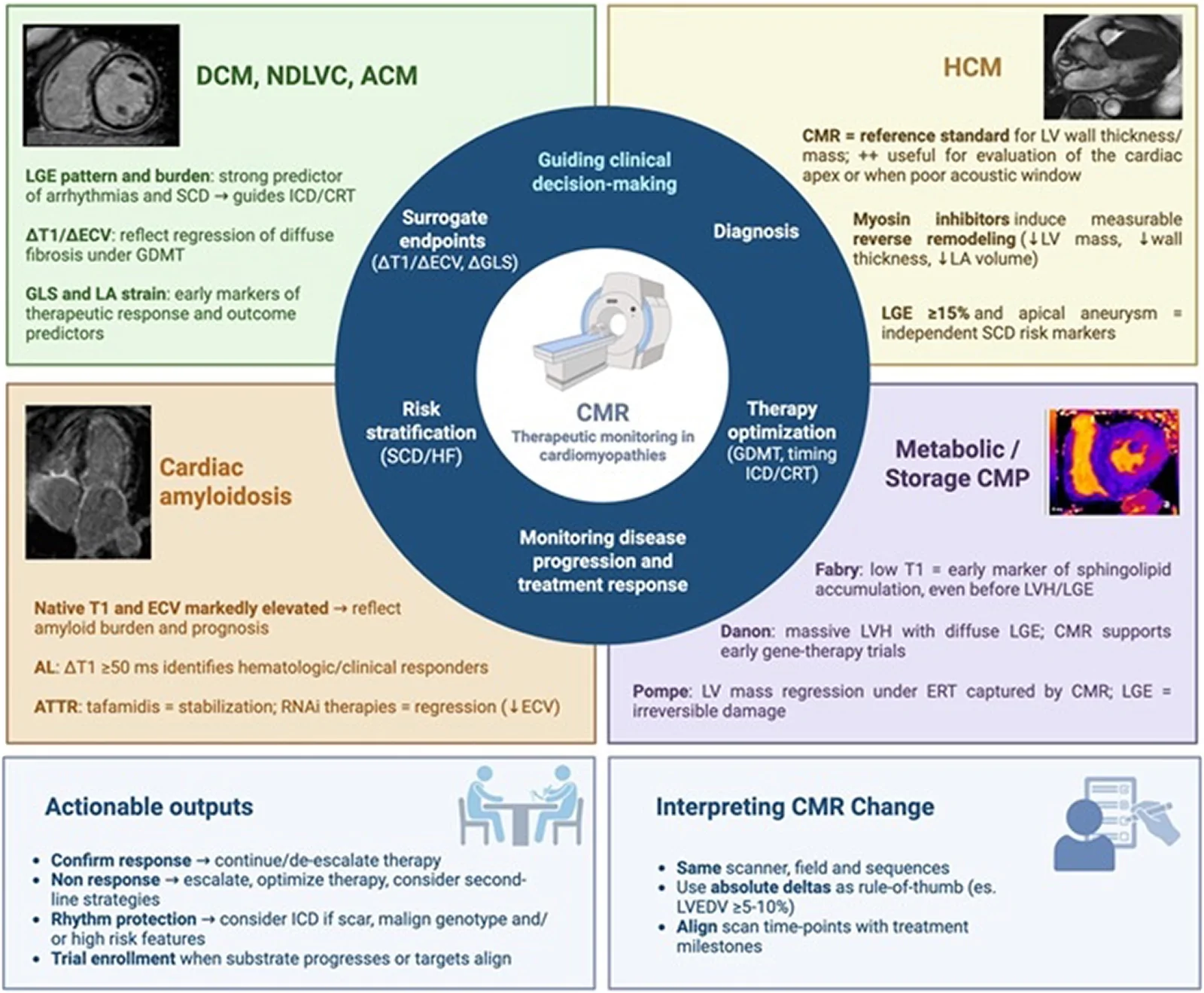
Cardiovascular magnetic resonance (CMR) for therapy monitoring in cardiomyopathies. This schematic figure illustrates the role of CMR in the therapeutic monitoring of cardiomyopathies. Imaging-derived surrogate markers, including changes in native T1, extracellular volume (ECV), and myocardial strain parameters such as global longitudinal strain (GLS), provide quantitative measures of myocardial remodelling and therapeutic efficacy. The figure highlights the application of CMR across major cardiomyopathy phenotypes. In dilated and arrhythmogenic cardiomyopathies, late gadolinium enhancement (LGE) burden and pattern predict arrhythmic risk and guide device therapy. In hypertrophic cardiomyopathy, CMR is the reference standard for left ventricular morphology and for assessing fibrosis and sudden cardiac death risk. In cardiac amyloidosis and metabolic/storage cardiomyopathies, tissue characterization with T1 mapping and ECV allows early detection, assessment of disease burden, and monitoring of treatment response. Abbreviations: ACM, arrhythmogenic cardiomyopathy; AL, light-chain amyloidosis; ATTR, transthyretin amyloidosis; CRT, cardiac resynchronization therapy; DCM, dilated cardiomyopathy; ERT, enzyme replacement therapy; GDMT, guideline-directed medical therapy; GLS, global longitudinal strain; HCM, hypertrophic cardiomyopathy; HF, heart failure; ICD, implantable cardioverter-defibrillator; LA, left atrium; LV, left ventricle; LVEDV, left ventricular end-diastolic volume; LVH, left ventricular hypertrophy; NDLVC, non-dilated left ventricular cardiomyopathy; RNAi, RNA interference; SCD, sudden cardiac death; T1, longitudinal relaxation time.

Cardiomyopathies are myocardial disorders characterized by structural and functional abnormalities not fully explained by abnormal loading conditions, coronary artery disease, valvular lesions, or congenital defects. The 2023 European Society of Cardiology Guidelines classify cardiomyopathies into five main phenotypes: dilated cardiomyopathy (DCM), non-dilated left ventricular cardiomyopathy (NDLVC), hypertrophic cardiomyopathy (HCM), arrhythmogenic right ventricular cardiomyopathy (ARVC), and restrictive cardiomyopathy (RCM).^1^ Therapeutic strategies for cardiomyopathies are evolving rapidly, and disease-specific agents are reshaping practice. These advances require imaging biomarkers that are sensitive, reproducible, and non-invasive to monitor structural, functional, and tissue-level responses to therapy.

Cardiovascular magnetic resonance (CMR) is the reference standard for comprehensive evaluation across cardiomyopathy phenotypes.^2^ Cine CMR yields highly reproducible measurements of biventricular volumes, mass, and biventricular ejection fraction (EF) for longitudinal assessment of remodelling. Beyond function, CMR provides non-invasive tissue characterization: late gadolinium enhancement (LGE) delineates focal replacement fibrosis, while quantitative mapping (native T1/T2) and extracellular volume (ECV) quantify diffuse interstitial involvement.^3^ In recent years, its role has expanded from diagnosis to longitudinal management—measuring reverse remodelling, stabilization, or progression under targeted therapies—and it supplies robust surrogate endpoints suitable for clinical trials.^4^

In this review, we appraise CMR as the pivotal modality for multidimensional evaluation of cardiomyopathies, with particular emphasis on therapeutic monitoring. The overarching role of CMR across cardiomyopathy phenotypes—from diagnosis and risk stratification to therapy optimization and longitudinal monitoring—is summarized in the *Graphical Abstract*. The main phenotype-specific CMR signatures discussed across cardiomyopathies are summarized in *Table 1*. Pertinent studies were identified through a targeted search of PubMed, Embase, and Medline (updated to March 2026) using combinations of keywords related to cardiomyopathies, CMR, tissue characterization, and therapy monitoring. Given the narrative and expert-based nature of this review, no formal systematic selection or appraisal criteria were applied.

**Table 1:** Phenotype-specific CMR signatures in cardiomyopathies

Phenotype	Typical LGE pattern	Native T1	T2/oedema	ECV	Perfusion/microvascular clues	Arrhythmic risk markers (CMR)	What to track on therapy
DCM	Mid-wall septal or patchy non-ischemic scar; generally non-regressive on follow-up.	Usually elevated; falls with reverse remodelling under GDMT.	Use when inflammation suspected; T2 mapping is an objective activity marker.	Elevated; associates with worse function/outcomes; small declines can parallel recovery.	Consider quantitative perfusion when ischemia/microvascular interventions are relevant.	Presence/extent and architecture of LGE (mid-wall) inform VA/SCD risk and device timing.	LV volumes/EF and LV mass; native T1/ECV trends; LGE burden/topology; GLS/LA strain.
NDLVC/ Left-dominant ACM	Mid-wall or subepicardial scar; ring-like patterns in some; fibro-fatty overlap in ACM spectrum.	‘Mapping-first’ changes (↓T1/↓ECV) precede geometry change and track recovery.	May signal ongoing inflammatory/immune injury in subsets.	Elevated ECV is a treat-to-target metric linked to outcomes.	Use quantitative perfusion where microvascular physiology is a therapeutic target.	LGE ≥9% LV mass predicts major arrhythmic events; ring-like LGE and specific genotypes add risk; emerging RV involvement ups risk.	Serial LGE quantification; native T1/ECV trends; RV size/function; escalate rhythm protection if scar progresses or RV involvement appears.
ARVC (right-dominant)	RV free-wall scar/fat; LV involvement may appear.	Mapping supportive; not primary driver.	T2 can help if inflammatory activity suspected.	Often not central; use selectively.	—	LV involvement (LGE/fat) quadruples arrhythmic risk; CMR-negative definite ARVC had no events in one series.	RV size/function stability on exercise restriction; new/expanding scar; watch for LV involvement.
HCM	Patchy mid-myocardial LGE in hypertrophied segments and at RV insertion points.	Elevated; abnormal even in genotype + /phenotype–; useful early biomarker.	Elevated T2 may mark active injury and higher arrhythmic risk in subsets.	Elevated; complements LGE for diffuse fibrosis.	Consider quantitative perfusion when ischemia/microvascular dysfunction is a management target.	LGE ≥15% LV mass doubles SCD risk; apical aneurysm adds risk.	Under myosin inhibitors: ↓LV mass index, ↓max wall thickness, ↓LA volume with preserved EF; track LGE extent and T1/ECV for substrate trends.
Amyloid CM	Diffuse subendocardial → transmural LGE (more often ATTR); difficulty nulling myocardium.	Markedly elevated (both AL and ATTR). In AL, ΔT1 ≥ 50 ms aligns with hematologic/organ response.	Usually not the primary marker.	Very high; stabilization on tafamidis (ATTR) vs. absolute declines with gene-silencing (e.g. patisiran).	—	Extent/pattern integrate with function for prognosis.	AL: falling native T1 with biomarker improvement; ATTR: flat T1/ECV/LGE on tafamidis = stabilization; ↓ECV on RNA-interference = regression.
Fabry disease	Mid-myocardial basal inferolateral LGE in advanced stages.	Reduced native T1 is characteristic and most sensitive early marker; may pseudo-normalize/rise with fibrosis.	Not primary.	Often near-normal until extracellular fibrosis develops.	—	Once LGE appears, risk and irreversibility rise; earlier therapy favoured.	Track native T1 (storage regression), LV wall thickness/mass, and emergence/progression of LGE; consider strain for early dysfunction.

AL, amyloid light-chain; ACM, arrhythmogenic cardiomyopathy; ARVC, arrhythmogenic right ventricular cardiomyopathy; ATTR, amyloid transthyretin; CMR, cardiovascular magnetic resonance; DCM, dilated cardiomyopathy; ECV, extracellular volume; EF, ejection fraction; GDMT, guideline-directed medical therapy; LA, left atrium; LGE, late gadolinium enhancement; LV, left ventricular; RV, right ventricular; SCD, sudden cardiac death; VA, ventricular arrhythmias.

## CMR methodologies for therapy monitoring

Balanced steady-state free precession (bSSFP) *cine imaging* remains the reference standard for quantifying biventricular volumes, myocardial mass, and global systolic function in cardiomyopathies. Its excellent test–retest reproducibility supports longitudinal assessment of disease trajectory and therapeutic response, particularly in dilated and hypertrophic phenotypes. In follow-up, changes that exceed inter-study variability, e.g. an absolute ≥5 percentage-point change in left ventricular EF (LVEF) or a ≥5% change in LV end-diastolic volume (LVEDV), have been associated with meaningful response to disease-modifying therapies, including beta-blockers, angiotensin-converting enzyme (ACE) inhibitors, and cardiac resynchronization therapy (CRT).^5^ Feature-tracking–derived strain (e.g. global longitudinal strain [GLS]) can reveal subclinical improvement earlier than LVEF and complements volumetric endpoints in therapy monitoring.

**LGE** is a cornerstone of CMR tissue characterization, enabling sensitive detection and semi-quantitative assessment of focal myocardial necrosis/fibrosis. Inversion-recovery gradient-echo imaging uses an inversion time to null normal (‘remote’) myocardium; phase-sensitive inversion recovery (PSIR) reduces sensitivity to TI mis-setting and yields robust visualization of both ischaemic and non-ischaemic scar patterns, aiding diagnostic differentiation.^6^ Although often a marker of irreversible replacement fibrosis, LGE can change in acute injury (e.g. myocarditis or recent myocardial infarction), where oedema resorption and infarct remodelling may reduce LGE extent in parallel with functional recovery.^7^

Quantitative native *T1 mapping* detects diffuse interstitial abnormalities arising from fibrosis, oedema/inflammation, or protein deposition. Fabry disease is a notable exception: native T1 is typically reduced due to intracellular sphingolipid accumulation and may pseudo-normalize or rise only when extracellular fibrosis develops.^8^ In amyloid transthyretin cardiomyopathy (ATTR-CM), native T1 is usually elevated and can reveal myocardial involvement even before overt hypertrophy, often outperforming LGE for early detection.^9^ Across phenotypes, native T1 carries diagnostic and prognostic value: in HCM, elevated T1 reflects diffuse fibrosis extending beyond hypertrophied segments and refines arrhythmic risk stratification^10^; in DCM, native T1 correlates with histological fibrosis, predicts progression, and adds incremental prognostic information over volumes and EF.^11^ Longitudinal data suggest native T1 can serve as a surrogate of reverse remodelling under guideline-directed therapy, capturing diffuse fibrosis regression that LGE may miss.

*T2 mapping* provides pixel-wise quantification of myocardial T2 relaxation time, a surrogate for tissue water content, and addresses many of the qualitative limitations of conventional T2-weighted imaging. In inflammatory cardiomyopathies, prolonged T2 reflects oedema from acute injury and often extends beyond regions of wall-motion abnormality or LGE, highlighting incremental diagnostic value.^12^ In HCM, elevated T2 has been associated with arrhythmic events, suggesting potential utility as a substrate marker within sudden cardiac death (SCD) risk assessment.^13^ In DCM and arrhythmogenic phenotypes, T2 abnormalities may indicate ongoing inflammatory or immune-mediated injury, supporting activity monitoring and higher-risk phenotyping. Overall, T2 mapping functions as an objective, dynamic marker of oedema and active injury, well suited to tracking therapeutic response.^14^

Post-contrast *ECV* quantifies diffuse interstitial remodelling driven by fibrosis, protein deposition, or metabolic storage, providing a global measure that correlates with collagen volume fraction and complements focal scar detection by LGE. Across phenotypes, ECV offers diagnostic, prognostic, and monitoring value: in DCM, elevated ECV reflects diffuse fibrosis and associates with impaired systolic function, arrhythmias, and adverse outcomes^15^; in HCM, ECV captures microscopic fibrosis beyond LGE-positive regions and refines risk stratification.^16^ In infiltrative disease such as ATTR-CM, ECV is typically markedly elevated and tracks disease burden, whereas in Fabry disease (an intracellular storage disorder) ECV may remain near-normal until extracellular fibrosis develops. Integrated into routine follow-up, ECV strengthens risk assessment and therapeutic monitoring by quantifying diffuse remodelling over time.

The principal CMR parameters used for serial therapy monitoring, together with pragmatic thresholds for repeated measurements, are summarized in *Table 2*.

**Table 2:** Cardiovascular magnetic resonance (CMR) parameters for therapy monitoring

Parameter	Modality/sequence	Minimal change with clinical relevance	Biological/clinical meaning	Considerations for repeated measurements
LVEF	Cine SSFP (short-axis stack)	≥5 percentage points	Global systolic function improvement or deterioration	Match vendor/field strength; identical segmentation rules; report papillary muscle treatment consistently
LV volumes indexed	Cine SSFP	EDV: ≥5–10% ESV: ≥10–15%	Reverse remodelling (↓ESV strongest) or adverse remodelling	Use same basal/apical slice definition; confirm end-diastolic/end-systolic frame picking method
LVMI	Cine SSFP	≥5–10%	Hypertrophy/storage regression (or progression)	Consistent basal slice selection; same contouring convention for papillary muscles
MWT	Cine SSFP (aligned to same planes)	≥2 mm	Myocardial de-thickening (e.g. under sarcomere/myosin inhibition)	Measure in diastole; replicate imaging planes; avoid partial-volume error
LV GLS	Feature-tracking on cine	≥2 absolute % points (e.g. −18% → −20%)	Early contractile improvement; sensitive to subendocardial change	Same software/version and ROI; re-check tracking and recontour if needed
LA reservoir strain	Feature-tracking on cine (2-/4-chamber)	≥4 absolute % points	Atrial compliance/loading improvement; complements LV metrics	Fixed views and frame rates; same vendor/software; report rhythm during scan
Native T1 (myocardial)	T1 mapping	Change exceeding site-specific repeatability (rule-of-thumb: ≥20–30 ms depending on field/sequence)	Diffuse myocardial disease activity/regression (fibrosis/storage/oedema)	Report sequence, field strength, vendor; ensure identical slice position; perform QC; avoid off-resonance/artifacts
ECV	Pre/post-contrast T1 + contemporaneous haematocrit	≥2 percentage points	Diffuse interstitial expansion/regression (fibrosis/amyloid)	Match contrast agent, dose, timing, T1 sequence, and haematocrit; avoid post-contrast timing drift
T2 mapping	T2 mapping	Change exceeding site-specific repeatability (rule-of-thumb: ≥2–3 ms at 1.5T)	Myocardial oedema/inflammation activity	Consistent slice position and heart rate; QC for artifacts; avoid sequence swaps between scans
LGE—presence/extent (% LV mass)	PSIR LGE (phase-sensitive)	New LGE or extent change ≥2–3 percentage points	Scar burden dynamics; substrate for arrhythmia/response	Standardize inversion/PSIR method; same voxel size and segmentation; prefer %LV mass for serials
LGE topology/pattern	PSIR LGE	Categorical change (e.g. new ring-like, mid-wall → transmural)	Substrate architecture shift with prognostic/risk implications	Classify patterns explicitly (mid-wall, subepicardial, ring-like, transmural); document RV involvement
Perfusion (quantitative)—stress MBF/MPR	Quantitative perfusion (pixel-wise MBF)	≥15% relative increase in stress MBF or MPR (site-specific)	Microvascular/ischemic improvement under therapy	Match stressor, dose, sequence, and analysis pipeline; ensure identical AIF method; avoid caffeine/vasodilator timing drift
RVEF and volumes	Cine SSFP (axial or short-axis RV set)	RVEF: ≥5 percentage points RVESV: ≥15%	RV function/remodelling (important in ARVC/ACM, pulmonary hypertension, amyloidosis)	Use a dedicated RV stack or standardized short-axis; consistent valve plane handling
RA area/volume	Cine SSFP (4-chamber)	≥10%	Atrial remodelling/load change	Consistent end-systolic frame and tracing landmarks; report rhythm

Thresholds are pragmatic ‘rule-of-thumb’ values intended to exceed typical inter-study variability.

AIF, arterial input function; CMR, cardiovascular magnetic resonance; ECV, extracellular volume; EDV, end-diastolic volume; ESV, end-systolic volume; GLS, global longitudinal strain; LA, left atrium; LGE, late gadolinium enhancement; LV, left ventricle; MBF, myocardial blood flow; MPR, myocardial perfusion reserve; MWT, maximal wall thickness; PSIR, phase-sensitive inversion recovery; QC, quality control; RA, right atrium; ROI, region of interest; RV, right ventricle; RVEF, right ventricular ejection fraction; RVESV, right ventricular end-systolic volume; SSFP, steady-state free precession; T1, longitudinal relaxation time; T2, transverse relaxation time.

## Specific disorders

Across the major phenotypes discussed below, expected CMR trajectories on therapy and the main safety checks during follow-up are summarized in *Table 3*.

**Table 3:** Therapy-by-phenotype: expected CMR trajectories and safety checks

Disease/Phenotype	Therapy class (examples)	Early CMR response markers (≈0–6 months)	Longer-term markers (≥6–12 months)	Safety monitoring on CMR	Flag for escalation/non-response
DCM	GDMT; device therapy as indicated (CRT/ICD)	↓LVESV (≥10–15%), ↑LVEF (≥5 pp), trend ↓native T1/ECV; improved LV GLS	Sustained reverse remodelling (↓LV volumes, ↑LVEF), stable or slowly changing LGE extent/topology; improved LA strain	Consistency of segmentation; watch for new LGE suggesting superimposed injury	No ESV reduction or falling LVEF; progressive dilation; new or expanding scar; frequent/complex ventricular arrhythmias
NDLVC/ALVC spectrum	HF therapy; arrhythmia prevention (ICD considered by risk); treat inflammatory drivers where present	‘Mapping-first’ improvement (↓ECV/normalized T1) before geometry changes; stable RV size/function	Stabilization or modest ↓LGE burden; maintained or improved LV/RV function; improved GLS	Monitor LGE topology (e.g. ring-like patterns), RV involvement, and any new scar	Rising scar burden or new ring-like LGE; emerging RV involvement; worsening function or recurrent VT/NSVT
HCM	Myosin inhibitors (e.g. mavacamten/aficamten) ± β-blocker; consider septal reduction if refractory	↓LV mass index, ↓max wall thickness (when present), improved LA strain; symptoms/gradients may improve in parallel	Sustained ↓mass/wall thickness with preserved EF; LGE usually stable; improved remodelling indices	Track LVEF to avoid excess negative inotropy (hold/titrate if EF < ∼50%); check LA size/strain	EF decline without clinical benefit; persistent obstruction with limited structural change → consider septal reduction pathway
ATTR-CM	TTR stabilizer (tafamidis); gene silencers (patisiran/vutrisiran/inotersen); supportive HF care	Tafamidis: stable T1/ECV/LGE; RNAi: early ↓ECV or T1 trends with stable function	Stabilized or reduced ECV; maintained function/strain; LGE pattern typically unchanged	Ensure serial ECV/T1 are truly comparable (dose/timing/sequence/Hct); watch for progression to transmural LGE	Rising ECV/T1 or worsening volumes/strain despite therapy; new effusions or rapid wall-thickening
AL-CM	Hematologic therapy (e.g. daratumumab-based chemo) ± ASCT; supportive HF care	↓Native T1 (e.g. ≥∼50 ms) paralleling hematologic response; stable volumes	↓ECV over time; improved or stable strain/function; reduced wall thickness rate of change	Consistency of mapping protocol; assess pericardial/pleural effusions and atrial size	No improvement or rising T1/ECV despite hematologic response; enlarging effusions; worsening function
Fabry disease	ERT or chaperone therapy	↑Native T1 towards normal (from low baseline), ↓LV mass index; stable or improved strain	Prevention of LGE expansion; continued ↓mass and stable function	Once LGE is established, reversal is limited—monitor for progression and wall thinning	Emergence or growth of LGE; rising wall thickness/mass despite therapy; declining strain
ARVC	Exercise restriction; β-blocker/antiarrhythmics; ablation; ICD as indicated	Stabilization of RV volumes and RVEF; no new LGE/fatty infiltration	Maintenance of RV function; absence of LV involvement	Monitor for new LV scar or dilation and progressive RV enlargement	Progressive RV dilation/dysfunction; new LV involvement; recurrent VT despite therapy
CRT responder tracking (subset of DCM)	Cardiac resynchronization therapy	↓LVESV ≥15% and/or ↑LVEF ≥5 pp by 6 months; improved synchrony indices (if available)	Sustained reverse remodelling and improved LA size/strain; stabilized or improved MR (if assessed)	Use identical short-axis coverage and contouring rules across serial scans	Failure to achieve reverse remodeling; rising volumes or unchanged ESV with persistent symptoms

‘Early’ vs. ‘longer-term’ time windows are pragmatic and may vary by therapy and disease severity. Percent/absolute cut-points are illustrative and should be interpreted against site-specific repeatability.

AL-CM, amyloid light-chain cardiomyopathy; ALVC, arrhythmogenic left ventricular cardiomyopathy; ARVC, arrhythmogenic right ventricular cardiomyopathy; ASCT, autologous stem-cell transplantation; ATTR-CA, amyloid transthyretin cardiomyopathy; CMR, cardiovascular magnetic resonance; CRT, cardiac resynchronization therapy; DCM, dilated cardiomyopathy; ECV, extracellular volume; EF, ejection fraction; ERT, enzyme replacement therapy; GDMT, guideline-directed medical therapy; GLS, global longitudinal strain; HCM, hypertrophic cardiomyopathy; Hct, haematocrit; HF, heart failure; ICD, implantable cardioverter-defibrillator; LA, left atrium/left atrial; LGE, late gadolinium enhancement; LV, left ventricle/left ventricular; LVEF, left ventricular ejection fraction; LVESV, left ventricular end-systolic volume; MR, mitral regurgitation; NSVT, non-sustained ventricular tachycardia; pp, percentage points; RNAi, RNA interference; RV, right ventricle/right ventricular; T1, longitudinal relaxation time (native T1); TTR, transthyretin; VT, ventricular tachycardia.

### DCM

DCM affects roughly 1 in 250 people.^17^ Serial CMR supplies quantifiable, mechanism-anchored endpoints that track reverse remodelling, detect non-response early, and refine event prediction while therapy is optimized. A minimal monitoring protocol typically comprises bSSFP cine imaging for chamber remodelling; LGE for scar burden and architecture; native T1 and ECV for diffuse interstitial fibrosis; T2 (with or without mapping) when inflammatory activity is suspected; and strain parameters, which may offer additional information on atrial–ventricular interaction, although evidence supporting their clinical use for this purpose remains limited. Practical protocols and quality safeguards now enable reproducible longitudinal measurements in DCM, with sequence selection, mapping standardization, and reporting tailored to serial follow-up. Cine-derived LV volumes, LVEF, and LV mass index (LVMI) remain the most responsive markers of pharmacologic reverse remodelling and form the backbone of treatment surveillance.

Under guideline-directed medical therapy (GDMT) for idiopathic DCM, longitudinal CMR demonstrates parallel improvements in LVEF and tissue phenotype: reductions in native T1 and ECV track with volumetric reverse remodelling and predict greater functional recovery. In a prospective cohort of 157 patients, reverse remodelling occurred in 31% over a median 14 months; responders were younger, had shorter disease duration, more advanced symptoms, lower baseline LVEF, and notably lacked focal fibrosis (LGE 15% vs. 43%; all *P* < 0.05–0.01). Tissue characterization showed reductions in native T1 (1303 ± 44 to 1245 ± 52 ms, *P* < 0.001), matrix volume (20.4 to 14.0 mL/m^2^, *P* < 0.001), and cell volume (52.5 to 37.7 mL/m^2^, *P* < 0.001); ECV (28.1% to 27.6%, *P* = 0.21) and T2 (51.1 to 50.8 ms, *P* = 0.74) were unchanged, though lower baselines predicted recovery. LGE did not regress, underscoring the irreversibility of replacement fibrosis; even responders retained residual abnormalities vs. healthy controls (native T1 1245 vs. 1205 ms, *P* < 0.05), supporting continued long-term therapy.^18^

Beyond absolute LGE burden, serial CMR characterizes scar architecture (core vs. border zone, proximity to conduction tissue), which shapes ventricular arrhythmia propensity. A ‘CMR-first’ device strategy uses follow-up scar maps to re-estimate SCD risk and time primary-prevention implantable cardiac defibrillator (ICD) discussions in parallel with medical uptitration.^19^ In this context, multimodality studies have shown that the combination of fibrosis imaging and perfusion–metabolism abnormalities provides incremental prognostic value, with coexisting LGE and perfusion–metabolism mismatch strongly associated with adverse outcomes in DCM.^20,21^

For patients with persistent symptoms and electrical dyssynchrony, CMR is increasingly central to optimizing and monitoring CRT. Pre-implant imaging identifies scar-free venous targets, characterizes mechanical dyssynchrony, and maps late-activated segments to guide lead delivery; tissue characterization adds prognostic value. In 101 non-ischaemic DCM patients undergoing CRT, non-responders had greater LGE burden (34.3% vs. 13.3%, *P* < 0.001), higher native T1 (1372 vs. 1337 ms, *P* = 0.033), higher T2 (46 vs. 42 ms, *P* < 0.001), and higher ECV (37% vs. 31%, *P* < 0.001). Predictive accuracy was greatest for LGE burden (area under the curve [AUC] 0.817) and ECV (AUC 0.808); thresholds of LGE >20% (odds ratio [OR] 0.15, 95% confidence interval [CI] 0.02–0.71) and ECV >34% (OR 0.13, 95% CI 0.01–0.78) identified patients unlikely to benefit. Post-implant, cine volumetry (ΔLV end-systolic volume [LVESV] > 15% or ΔLVEF >5%) remains the accepted remodelling endpoint, while serial LGE and strain corroborate reverse remodelling or signal persistent non-response—positioning CMR as a comprehensive tool for longitudinal CRT management.^22^

In patients carrying cardiac implantable electronic devices (CIEDs), serial CMR assessment presents additional technical challenges related to device-related artefacts and sequence limitations. Although contemporary magnetic resonance (MR)-conditional devices allow safe imaging with a high overall diagnostic yield, image quality may be variably impaired—particularly in anterior and anteroseptal segments and with balanced SSFP cine sequences—while ICDs typically generate more extensive artefacts than pacemakers.^23^ Despite these limitations, CMR after CRT implantation remains feasible. Practical strategies to optimize image quality include the use of gradient-echo cine sequences instead of SSFP when artefacts are significant, implementation of wideband LGE techniques to reduce hyperintensity artefacts, and consideration of arm positioning (e.g. arm-raised imaging) to increase generator–heart distance. Device-related factors such as generator location (right-sided systems when feasible) and careful pre-scan programming into MRI-safe modes are also important. When interpreting serial changes, particular caution is required for LGE assessment in affected segments, while volumetric and strain parameters are generally more robust and can still provide clinically meaningful information for therapy monitoring and CRT optimization.^24^

GLS enhances sensitivity for early response before LVEF changes are apparent. A meta-analysis of >1200 non-ischaemic patients confirmed impaired GLS as a significant predictor of mortality (hazard ratio [HR] 1.27, 95% CI 1.10–1.46, *P* = 0.001), with an optimal threshold around −12.5% to −13.5%.^25^

Left atrial (LA) deformation adds complementary prognostic information. In 488 patients followed for a median 6 years, LA conduit strain predicted adverse outcomes (HR 3.65, 95% CI 2.01–6.64, *P* < 0.001); reservoir strain was also significant, whereas booster strain was not. LA strain improved discrimination (C-index 0.62–0.70, *P* < 0.001) and remained prognostic after adjustment for LVEF, GLS, and LGE, with highest risk when fibrosis coexisted with severely reduced conduit strain (<11%; log-rank *P* < 0.001).^26^

Integration of strain with tissue characterization enriches therapeutic monitoring. Native T1 and ECV respond to the antifibrotic effects of GDMT and, with established test–retest characteristics and acquisition/analysis guidance, support ΔT1 and ΔECV as intermediate endpoints alongside volumetric reverse remodelling. Serial ECV, rather than single time points, most faithfully captures dynamic interstitial remodelling under therapy and correlates with functional recovery and outcomes, enabling ‘treat-to-target’ strategies that pair ECV with cine/strain. A multiparametric CMR approach improves early identification of responders and flags patients who may require escalation (advanced HF therapies, device implantation, or trial enrolment).

### Non-dilated left ventricular cardiomyopathy (NDLVC)

According to the ESC classification, NDLVC and ARVC are defined as distinct phenotypic categories. However, in clinical practice, NDLVC often represents a working diagnosis that may encompass heterogeneous substrates, including early or left-dominant forms within the arrhythmogenic cardiomyopathy (ACM) spectrum. ACM is increasingly recognized as a broader entity including right-dominant (ARVC), left-dominant, and biventricular forms.^27^ For clarity, NDLVC and ACM are discussed separately in this review, while acknowledging areas of phenotypic and pathophysiological overlap.

NDLVC is defined by the presence of LV systolic dysfunction in the absence of ventricular dilation and/or non-ischaemic myocardial fibrosis detectable by CMR.^1^ Within this spectrum, CMR functions as the central tool not only for diagnosing NDLVC but also for therapy monitoring, translating tissue and remodelling changes into actionable management.

A mapping-first longitudinal strategy detects treatment effects before gross geometric change. Decreases in native T1 and ECV track with recovery of LVEF and GLS and outperform EF alone for early responder classification, supporting T1/ECV as intermediate therapeutic endpoints.^28^ Current diagnostic pathways explicitly embed LGE and quantitative mapping as decision nodes for therapy escalation and rhythm protection.^29^

Fibrosis burden measured by LGE is the dominant arrhythmic substrate to follow over time in NDLVC. In a multicentre cohort (*n* = 225), LGE was prevalent (82%) and strongly prognostic; LGE ≥9% of LV mass best predicted major arrhythmic events (HR 3.74, 95% CI 1.19–11.77; *P* = 0.024). Risk rose further when extensive LGE coexisted with fibro-fatty replacement (HR 4.73, 95% CI 1.44–15.52; *P* = 0.010), whereas fibro-fatty change alone was not prognostic. Over a median of 3.3 years, 5% experienced SCD/ventricular fibrillation (VF)/sustained ventricular tachycardia (VT), and patients meeting 2024 European Task Force criteria for definite/borderline arrhythmogenic LV or biventricular cardiomyopathy (ALVC/ABVC) had shorter arrhythmia-free survival (HR 5.68, 95% CI 1.27–25.38; *P* = 0.023). These data anchor monitoring to serial LGE quantification, with intensification of rhythm protection when scar progresses or coexists with fibro-fatty change.^30^

Genotype and scar topology further refine longitudinal targets. In a large prospective cohort integrating CMR and genetics (*n* = 616; 462 DCM, 154 NDLVC), NDLVC showed lower rates of LV reverse remodelling than DCM (41% vs. 54%), while ring-like LGE (HR 2.35, 95% CI 1.29–4.26) and arrhythmogenic genotypes (*LMNA, FLNC, DSP, RBM20*; HR 2.26, 95% CI 1.40–3.66) independently predicted adverse outcomes—signals to track during follow-up.^31^ In the DERIVATE cohort, overall NDLVC had fewer major arrhythmic events than DCM (HR 0.45, 95% CI 0.22–0.93), but risk diverged by CMR subtype: hypokinetic NDLVC carried substantially lower event rates (HR 10.7 for DCM vs. hypokinetic NDLVC; *P* = 0.003), whereas fibrotic NDLVC resembled LGE-positive DCM. Mid-wall LGE independently predicted MACE (HR 6.7, 95% CI 1.33–33.67), suggesting that persistence or expansion of mid-wall scar warrants tighter surveillance and earlier device consideration, whereas an LGE-negative pattern supports ongoing guideline-directed therapy with an expectation of a more favourable course.^32^

Beyond overall LGE burden and topology, emerging data highlight the importance of active myocardial injury in refining arrhythmic risk in NDLVC. In a large multicentre study including 337 patients with NDLVC, myocardial inflammation—detected by CMR and/or endomyocardial biopsy—was associated with a particularly high risk (HR 15.7, 95% CI 6.1–40.3), supporting the concept that dynamic disease activity may contribute to arrhythmic vulnerability beyond fixed fibrotic substrate.^33^ Notably, these findings reinforce the value of serial CMR not only for quantifying fibrosis burden, but also for characterizing scar distribution and identifying markers of ongoing injury, which may have direct implications for risk stratification and timing of ICD implantation.

### Arrhythmogenic cardiomyopathy (ACM)

ACM encompasses a spectrum of inherited myocardial diseases characterized by progressive fibro-fatty replacement and a high propensity for ventricular arrhythmias, in which CMR plays a central role in defining disease expression, refining risk stratification, and guiding longitudinal management.

Importantly, CMR findings should not be interpreted in isolation, but integrated with ECG, arrhythmic, familial, and genetic data, both for diagnostic adjudication and for risk stratification across the ACM spectrum.

In suspected ACM, CMR protocols should include RV-dedicated cine views in addition to conventional long- and short-axis acquisitions, as focused RV inflow, RV outflow tract, and transaxial planes may improve detection of regional akinesia, dyskinesia, bulging, or microaneurysms.^28^

In right-dominant ARVC, lifestyle intervention is disease-modifying. Imaging phenotype directs management intensity: LV involvement (LGE and/or fat) quadruples major arrhythmic risk (HR 4.2, 95% CI 2.1–8.4; *P* = 0.0001) with markedly worse event-free survival vs. RV-only or CMR-negative disease (log-rank *P* < 0.0001). Notably, no events occurred in definite ARVC with entirely negative CMR, underscoring the value of serial LGE/mapping for early ICD consideration and stricter exercise limits; fat-predominant change alone is not prognostic and should not dictate therapy.^34^

Right ventricular strain analysis further refines phenotypic characterization in ARVC, with feature-tracking–derived RV global longitudinal strain showing high diagnostic accuracy and incremental value over conventional CMR criteria, particularly in patients with borderline disease.^35^

In ALVC/ABVC, non-ischaemic LV LGE—typically subepicardial and often involving the inferolateral wall, sometimes with a circumferential ‘ring-like’ pattern—may precede overt wall-motion abnormalities or global systolic dysfunction, underscoring the added value of CMR for early phenotypic characterization.^28^ Within ALVC, monitoring must account for both LV scar/fat burden and RV involvement to calibrate protection. In a 53-patient series, LV fibro-fatty infiltration and systolic dysfunction were typical; 32% had RV fibro-fatty change with mild–moderate RV dysfunction. Scar/fat burden correlated inversely with LVEF, and RV involvement signalled poorer transplant-free survival. A pragmatic RV target emerged: RVEF 44% identified RV involvement with excellent discrimination (AUC 0.994; sensitivity 100%, specificity 94%), supporting serial RV functional assessment as a decision point for reinforced exercise restriction and earlier ICD where LV scar coexists.^36^ Although not routinely required in all ACM studies, oedema-sensitive sequences and T2 mapping may be particularly informative in patients presenting with ‘hot-phase’ disease, where acute myocardial injury and inflammation can be captured by CMR and may contribute to dynamic risk assessment.^28^

Risk in ACM is dynamic and benefits from periodic re-estimation as imaging data accrue. Contemporary guidance emphasizes combining morphology (evolving LV/RV size and function), tissue characterization (fibrosis burden/pattern on LGE and mapping), ECG/arrhythmia markers, and genotype. This favours a programmatic, serial-CMR pathway: protection is escalated when scar progresses or RV involvement emerges and de-intensified when the substrate remains stable.^37^

### HCM

HCM, the most common inherited cardiac disease (1:200–1:500), results from sarcomeric mutations and is characterized by otherwise unexplained LV hypertrophy with highly variable expression, from asymptomatic carriage to obstruction, arrhythmias, heart failure, or SCD.^38^ Echocardiography remains first line, but CMR is the reference modality for serial, quantitative monitoring. Its tomographic coverage and high spatial resolution enable precise quantification of LVMI, maximal wall thickness (MWT), and chamber volumes, metrics central to documenting reverse remodelling and decongestion over time.^39^

**Figure 1:**
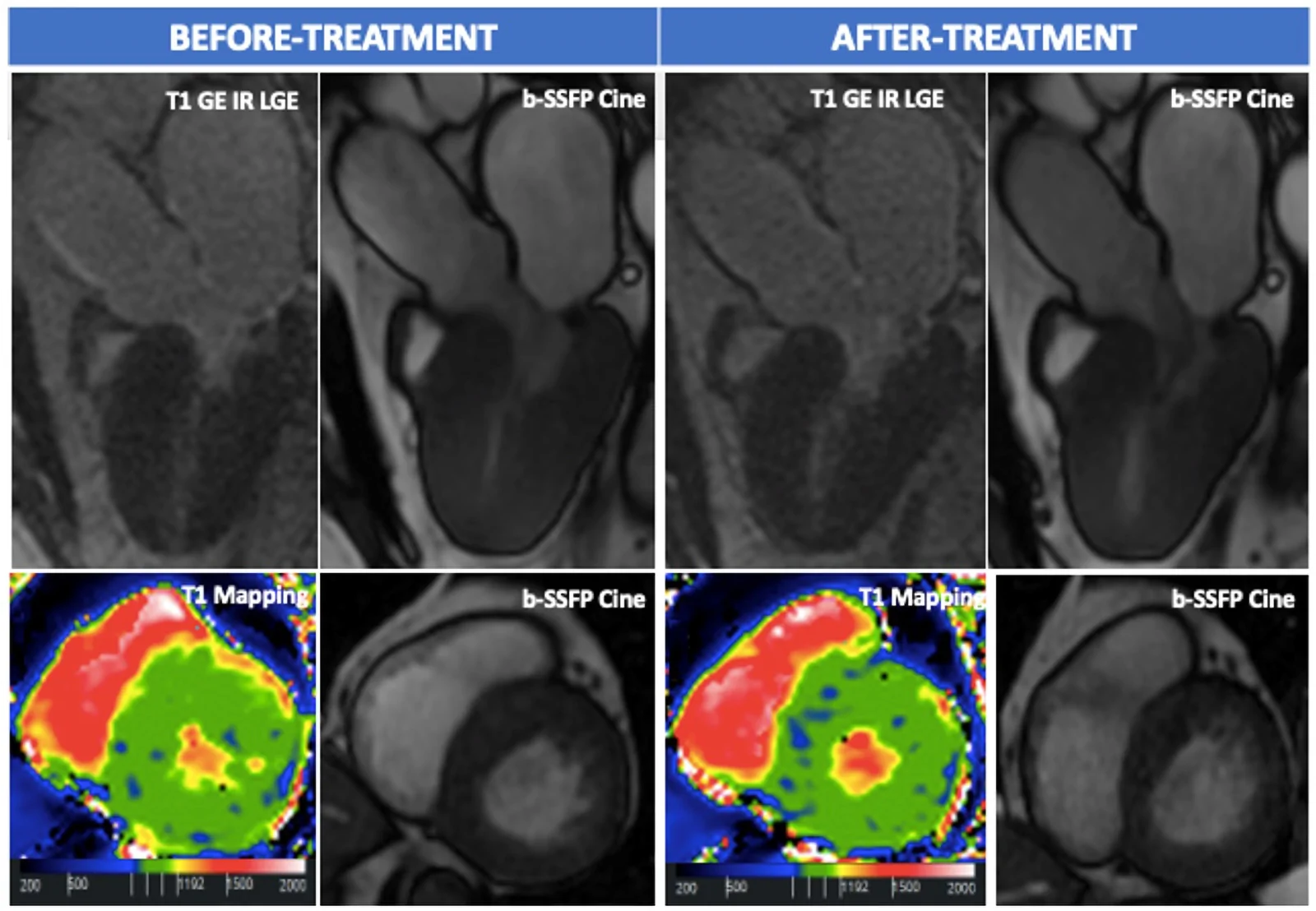
Cardiac magnetic resonance (CMR) images in a patient with hypertrophic cardiomyopathy (HCM) at baseline (pre-treatment) and after 1 year of treatment with aficamten (post-treatment). Follow-up CMR demonstrates a reduction in left ventricular (LV) mass (107 to 94 g) and maximal wall thickness (19 to 17 mm), consistent with LV reverse remodelling during myosin inhibitor therapy. Native T1 relaxation time decreased from an elevated baseline of 1103 to 980 ms following aficamten therapy (institutional normal reference range: 996–1092 ms).

For pharmacologic therapy, particularly cardiac myosin inhibitors (CMIs), which target sarcomere hypercontractility by reducing actin–myosin cross-bridge formation, CMR provides mechanism-anchored endpoints of disease modification. In EXPLORER-HCM, 30 weeks of mavacamten reduced LVMI, MWT, and LA volume while preserving systolic function (no CMR-derived EF <50%), defining a characteristic remodelling signature under CMIs.^40^ In routine care, repeat CMR at treatment milestones confirms structural response (decreases in LVMI, MWT, LA volume) and evaluates safety (stable EF), helping distinguish true structural change from transient load effects. *Figure 1* illustrates CMR evidence of LV reverse remodelling after one year of aficamten therapy in a patient with HCM. Beyond pharmacotherapy, CMR is integral to planning and auditing septal reduction: pre-procedural scans delineate target hypertrophy and mitral–subvalvular anatomy to select myectomy vs. alcohol septal ablation, while post-procedural scans characterize iatrogenic scar (typically more discrete after myectomy, more heterogeneous after alcohol ablation) and reassess mass, outflow geometry, and atrial remodelling—parameters linked to symptom relief and subsequent management.^41^

Tissue characterization refines risk and captures treatment effects that precede gross geometric change. LGE is present in 40–80% of patients (classically patchy mid-myocardial enhancement in hypertrophied segments or at RV insertion points) and extent, rather than presence alone, is prognostically decisive: LGE ≥15% of LV mass doubles SCD risk and tracks progression towards ‘end-stage’ HCM with wall thinning, LV dilation, and reduced EF.^42^ Parametric mapping adds sensitivity to diffuse interstitial remodelling: native T1 and ECV are elevated in overt HCM and often abnormal in genotype-positive/phenotype-negative individuals, supporting their role as early biomarkers that may show favourable trends before volumetric indices change substantially.^43^ These biomarkers are embedded in contemporary decision frameworks: CMR-derived MWT underpins the European Society of Cardiology (ESC) HCM Risk-SCD model, and the 2024 American guidelines incorporate CMR features (apical aneurysm and LGE ≥15%) as independent risk markers to inform ICD discussions and rhythm-surveillance intensity.^1,44^

Recent data from the prospective NHLBI HCM Registry further strengthen the prognostic role of CMR-derived structural and tissue markers in HCM.^45^ In 2698 patients followed for a mean of 6.9 years, the primary composite outcome was independently associated with LGE extent expressed as percentage of LV mass (HR 1.86 per 10-unit increase), LV mass index (HR 1.09 per 10-unit increase), LV end-systolic volume index, heart failure history, and N-terminal pro B-type natriuretic peptide (NT-proBNP). Notably, an LGE burden ≥9% of LV mass was associated with a substantially higher primary event rate, while the model for sudden cardiac death/nonfatal ventricular arrhythmias included LGE%, LV mass index, LVEF, and NT-proBNP. These findings complement guideline-based use of extensive LGE, but also suggest that lower LGE thresholds and LV mass index may add prognostic information for longitudinal risk recalibration and may be useful CMR endpoints when monitoring disease progression or response to disease-modifying therapy.

Interpretation of longitudinal change emphasizes measurement precision and biological coherence. Greater weight is placed on absolute deltas exceeding the laboratory’s minimal detectable change and on concordant signals across modalities. Under CMIs, a pattern of falling LVMI, reduced MWT and LA volume with preserved EF indicates reverse remodelling; LGE typically evolves slowly, so clear increases in scar burden (beyond segmentation variability) or emergence of an apical aneurysm warrant intensified protection. Meaningful downward shifts in native T1/ECV—when beyond site-specific repeatability—support antifibrotic effect and can validate early therapeutic benefit.^40^ Reproducible monitoring rests on consistent scanners and sequences, explicit reporting of field strength and mapping approach, and stable contrast dose/timing for ECV; aligning scan time points with therapeutic milestones (e.g. CMI initiation/titration, pre-/post-septal reduction) and with clinical triggers (arrhythmic symptoms, biomarker shifts) enhances interpretability.

In summary, CMR has progressed from adjunctive diagnosis to the central tool for therapy monitoring in HCM, quantifying reverse remodelling under CMIs, auditing anatomical and tissue-level results after septal reduction, and recalibrating arrhythmic risk through fibrosis phenotyping and mapping-based indices, thereby supplying actionable endpoints that guide the timing and intensity of medical therapy, intervention, and device implantation.

### Amyloid cardiomyopathy

Amyloid cardiomyopathy results from extracellular deposition of amyloid fibrils, and CMR is now the reference modality for diagnosis and longitudinal monitoring.^46^ Quantitative biomarkers track myocardial infiltration and its evolution under treatment, complementing NT-proBNP and providing mechanism-linked endpoints for clinical decisions.

**Figure 2:**
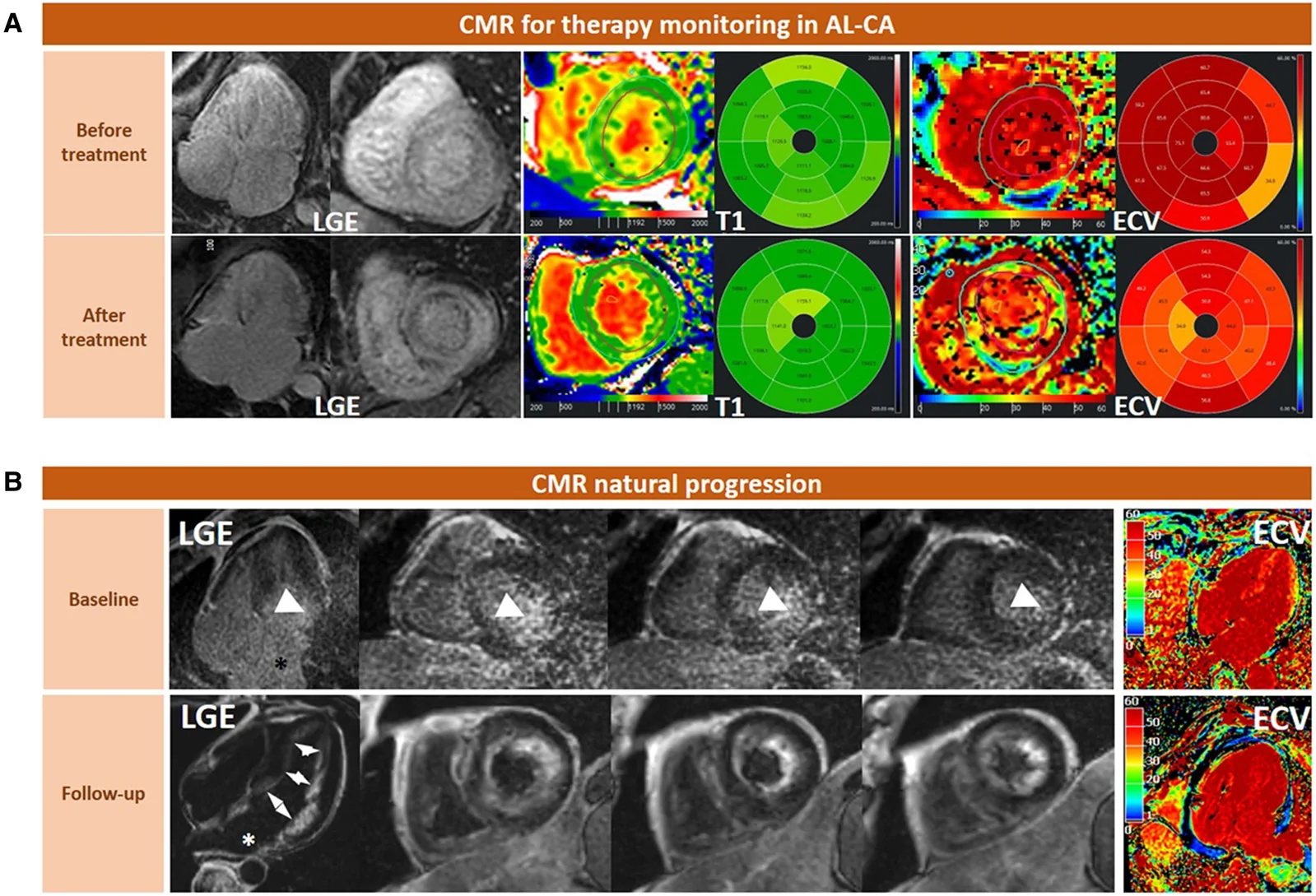
Cardiovascular magnetic resonance (CMR) for therapy monitoring in light chain cardiac amyloidosis (AL-CA). (*Panel A*): CMR images from a patient with AL-CA acquired at baseline and after treatment with daratumumab–cyclophosphamide–bortezomib–dexamethasone (Dara-CyBorD). At baseline (top panels), late gadolinium enhancement (LGE) images showed diffuse myocardial hyperintensity in both ventricles and both atria (hardly distinguishable from bloodpool hyperintensity), consistent with high myocardial gadolinium uptake due to diffuse amyloid infiltration. Native T1 mapping (global native T1 1097 ms) and extracellular (ECV) mapping (global ECV 62%) were very high, confirming myocardial amyloid infiltration. After two years of treatment (bottom panels), LGE images still showed diffuse amyloid infiltration, while native T1 mapping (global native T1 1079 ms) and ECV mapping (globale ECV 45%) were significantly improved compared to baseline, consistent with a lower myocardial amyloid burden after therapy. (*Panel B*): CMR images from a patient with multiple myeloma showing the natural progression of AL-CA. Baseline images (top panels) demonstrated limited subendocardial LGE in the inferolateral wall (arrows), as well as a slightly increased myocardial ECV (global value 38%). During follow-up, the patient discontinued his medical therapy, and his follow-up CMR (bottom panels) showed significant progression of myocardial amyloid infiltration, including diffuse subendocardial LGE (arrowheads) and further increased myocardial ECV (global value 52%). Please also note that, compared to baseline LGE images (black asterisk), follow-up LGE images presented marked bloodpool hypointensity (white asterisk), a hallmark of advanced AL-CA.

In *amyloid light-chain cardiomyopathy (AL-CM***)**, CMR offers a sensitive readout of haematologic and organ response. Serial native T1 mapping (contrast-free) tracks myocardial change with therapy: in 221 patients, a ≥50 ms fall identified haematologic responders and aligned with NT-proBNP reduction, regression of LV wall thickness and ECV, and improved strain, whereas a ≥50 ms rise signalled poor haematologic response, biomarker worsening, LV dysfunction, and higher mortality (HR 2.41, 95% CI 1.36–4.27).^47^  *Figure 2* demonstrates improvement in native T1 mapping values on follow-up CMR after therapy in a patient with AL-CM.

In *ATTR-CM*, CMR typically shows diffuse subendocardial or transmural LGE, markedly elevated native T1, and high ECV.^48^ Given slower progression than AL, stability of ECV/T1/LGE on disease-modifying therapy is a clinically meaningful success signal; upward trends indicate ongoing deposition. The most robust evidence is with tafamidis: a meta-analysis across ∼30 studies (∼3000 patients) showed reduced all-cause and cardiovascular mortality, preserved functional capacity, and attenuation of CMR-derived progression. On therapy, LVEF and RV function remain stable and ECV does not rise; native T1 and LVMI likewise stabilize, mirroring observational series that document maintenance of LV volumes, mass, and ECV on treatment vs. progressive decline off therapy.^49^ In contrast, RNA-interference therapies demonstrate regression: in variant ATTR-CM, 12 months of patisiran reduced myocardial ECV by 6.2% (95% CI −9.5 to −3.0; *P* = 0.001), with concurrent NT-proBNP reductions, improved 6-minute walk distance, and a median 19.6% fall in bone-scintigraphy uptake—supporting true substrate regression.^50^ Next-generation strategies aim for durable suppression or elimination of amyloidogenic substrate; for example, the phase 3 MAGNITUDE trial (NCT06128629) is testing *in vivo* CRISPR–Cas9 TTR editing (NTLA-2001), with ECV and functional CMR metrics prespecified as key readouts. Immune-mediated clearance remains an intriguing possibility: a small series reported near-complete ECV regression and normalization of structure and function in ATTR-CM, associated with circulating polyclonal IgG against ATTR fibrils.^51^

Longitudinal interpretation hinges on absolute deltas that exceed site-specific repeatability and on concordance with clinical/biochemical data. In AL-CM, a ≥50 ms fall in native T1 together with improving NT-proBNP and strain supports therapeutic response, with the expectation that extracardiac ECV may regress earlier than myocardial ECV. In ATTR-CM, flat ECV/T1/LGE on tafamidis signals stabilization, whereas an absolute ECV decline with RNA-interference therapy indicates substrate reduction. Rising ECV/T1, expanding LGE, or worsening strain/volumes despite treatment should prompt reassessment of adherence and regimen, consideration of escalation or alternative agents, and evaluation for trial enrolment.

Quantitative perfusion CMR may provide incremental insight into early disease processes in cardiac amyloidosis, particularly by capturing microvascular dysfunction before overt structural abnormalities become evident.^21^ Stress MBF and MBR have been identified as early disease markers, particularly in patients with subclinical amyloid infiltration (elevated ECV without overt LGE). These perfusion parameters correlate with native T1 mapping but not with T2, supporting the role of MPR as a functional marker of early amyloid-related microvascular dysfunction.^52^

Reproducible monitoring relies on consistent scanners and sequences, explicit reporting of field strength and mapping approach, stable contrast dose/timing for ECV with contemporaneous haematocrit (or declared synthetic haematocrit), and alignment of scan time points with treatment milestones (e.g. post-cycle AL assessments; tafamidis or RNA-interference initiation and 6–12-month follow-ups).

In summary, CMR has become the backbone of therapy monitoring in amyloid CM: native T1, ECV, and LGE provide quantitative, mechanistically anchored biomarkers that verify stabilization with TTR stabilizers, demonstrate regression with gene-silencing therapies, and integrate systemic response via multiorgan ECV, supporting timely treatment adaptation, robust prognostication, and individualized care.

### Metabolic and storage cardiomyopathies

Metabolic and storage cardiomyopathies are genetically determined disorders with intracellular substrate accumulation that drives remodelling, hypertrophy, and fibrosis, often mimicking hypertrophic or dilated phenocopies. CMR offers reproducible morpho-functional and tissue characterization for early recognition and therapy monitoring as enzyme replacement, pharmacologic chaperones, and gene therapies expand.

**Figure 3:**
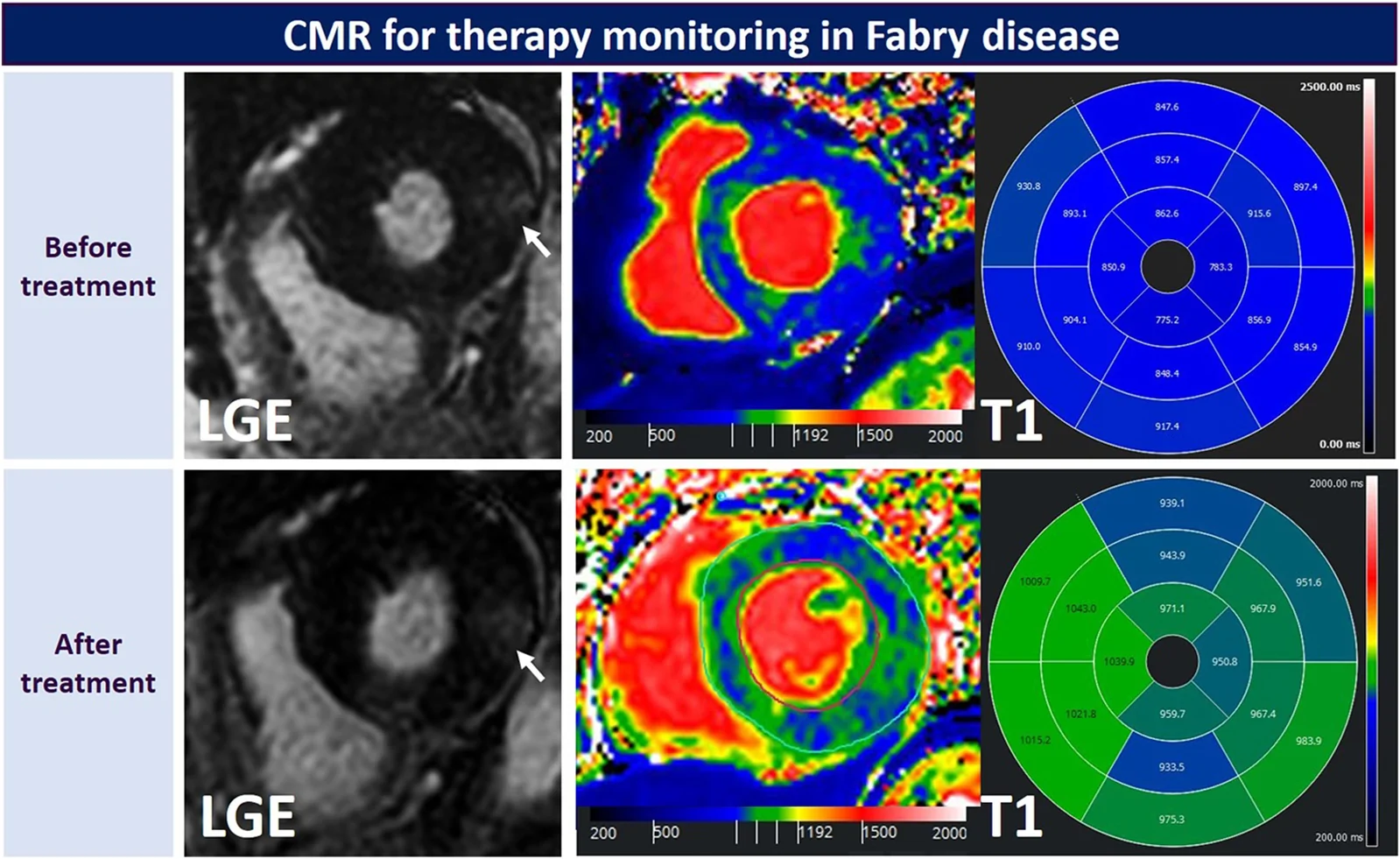
Cardiovascular magnetic resonance (CMR) for therapy monitoring in Fabry disease. A middle-aged lady with Fabry disease underwent CMR at baseline and after 2 years of treatment with enzyme replacement therapy (ERT). Baseline CMR (top panels) demonstrated mild intramyocardial late gadolinium enhancement (LGE) in the basal inferolateral left ventricular wall (arrow), as well as a typical diffuse reduction of native T1 mapping values (global native T1 879 ms). After therapy (bottom panels), the inferolateral LGE area remained unchanged (arrow), but native T1 mapping values significantly increased to nearly normal values (global native T1 961 ms).

In *Fabry disease*, systemic globotriaosylceramide (Gb3) accumulation commonly causes non-obstructive LV hypertrophy and can resemble sarcomeric HCM, with marked heterogeneity in female heterozygotes.^53^ Native T1 mapping is the earliest and most sensitive marker: Fabry uniquely shows reduced native T1, often starting basally inferolateral, preceding LV hypertrophy and LGE. LGE (typically mid-myocardial basal inferolateral) denotes replacement fibrosis, worse prognosis, and limited reversibility, emphasizing early treatment. Mapping remains valuable in atypical/non-hypertrophic phenotypes. *Figure 3* shows CMR changes in Fabry disease before and after Enzyme Replacement Therapy (ERT), with improvement in native T1 values and stable LGE.

In *Danon disease*, Lysosome-Associated Membrane Protein (LAMP)2-related autophagy defects cause early, aggressive cardiomyopathy with concentric hypertrophy and high arrhythmic risk. CMR typically shows massive hypertrophy with extensive LGE, elevated native T1, and markedly impaired strain despite preserved EF.^54^ With adeno-associated virus LAMP2B gene therapy entering early trials, CMR serves as a primary efficacy/safety readout to detect stabilization of LV mass and scar before EF changes; T2 mapping may help gauge post-therapy inflammation.^55^

Lysosomal glycogen accumulation is the hallmark of *Pompe disease*. Infantile-onset disease presents with LV hypertrophy; late-onset forms have milder but progressive involvement. LVMI is the most responsive metric under ERT, often normalizing within months on long-term follow-up, though arrhythmias can occur. LGE suggests irreversible injury, while native T1/T2 mapping is under study to track fibrosis and inflammation^56^; future directions include multiparametric and 4D-flow CMR for earlier, mechanism-linked endpoints.^57^

## Practical considerations for serial monitoring and interpreting change over time

Reliable therapy monitoring in serial CMR rests on protocol consistency (ideally the same system, field strength, coils, and pulse-sequence implementations) with documentation of key parameters such as breath-hold vs. free-breathing, slice thickness, and temporal resolution. Importantly, vendor-specific implementations and field strength (1.5T vs. 3T) may significantly influence absolute T1 and T2 values, and should therefore be kept constant across serial examinations whenever possible or explicitly accounted for when interpreting longitudinal changes. Routine quality assurance with phantoms and periodic repeatability checks helps identify drift.

For mapping, vendor- and sequence-specific reference ranges apply, and reports specify the method, acquisition plane, and heart-rate (HR) influence. Native T1 and T2 values may be influenced by HR, which is particularly relevant in the context of therapy monitoring, as many treatments reduce HR; therefore, longitudinal changes in mapping parameters should be interpreted with caution and not assumed to reflect tissue changes alone. Practical mitigation strategies include maintaining similar physiological conditions across serial scans, reporting HR at the time of acquisition, and—when available—using sequence implementations less sensitive to HR (e.g. longer recovery periods or HR–independent sampling schemes).^58^ In addition, consistency in sequence type and acquisition parameters across time points is essential to minimize HR–related variability.

For ECV, contemporaneous haematocrit, stable contrast agent/dose, and consistent post-contrast timing are preferred, while mixing field strengths across time points is minimized. When contemporaneous haematocrit is unavailable, synthetic haematocrit–derived ECV may be used as a practical alternative, although caution is warranted in patients with extreme haematocrit values or advanced cardiac dysfunction.^59^ A multiparametric strategy strengthens biological plausibility (volumes/EF for remodelling, LGE for focal replacement fibrosis, native T1/ECV for diffuse interstitium, and T2 for oedema/activity) with strain offering an early functional readout; in selected phenotypes (e.g. HCM, amyloidosis), stress perfusion/perfusion mapping informs microvascular change.

Reporting typically specifies absolute vs. relative deltas, includes measurement uncertainty, and links interpretations to clinical endpoints and treatment milestones. When longitudinal response is assessed, changes that exceed the laboratory’s minimal detectable change (MDC) and show cross-modal biological coherence carry greater weight. Practical guardrails include an absolute ≥5 percentage-point shift in LVEF or a ≥5–10% change in LV volumes as likely meaningful; for LGE, changes clearly beyond segmentation/test–retest variability interpreted alongside symptoms, biomarkers, and mapping; for native T1/T2, reliance on sequence-, field-, and vendor-specific reference ranges with site-validated repeatability, with acquisition method and heart rate specified; and for ECV, contemporaneous haematocrit and consistent post-contrast timing, with absolute shifts generally more interpretable than relative percentages. Interpretation remains integrated with clinical context. Improvements in oedema (T2) may precede or exceed changes in fibrosis (T1/ECV) and volumes. Protocols commonly pre-specify response thresholds and report confidence intervals, and single time-point fluctuations near the MDC are treated cautiously.

Finally, in research settings and clinical trials, the use of core laboratories and standardized acquisition and post-processing pipelines further improves reproducibility and reduces inter-site variability, which is critical for the use of CMR-derived biomarkers as surrogate endpoints.

Selected LGE and mapping thresholds that inform risk stratification or response assessment on serial follow-up are summarized in *Table 4*. It should be noted that while some imaging thresholds are supported by robust evidence and incorporated into guideline recommendations, others are derived from cohort studies or represent pragmatic, context-dependent values, and should therefore be interpreted within the clinical setting and in conjunction with serial changes over time.

**Table 4:** LGE/mapping thresholds that inform risk or response

Context/disease	Imaging threshold or pattern	What it predicts	How to act on serial follow-up
HCM	LGE ≥15% of LV mass	Higher SCD/VA risk; adverse prognosis	Lower the threshold for ICD consideration (per overall risk model); if LGE expands, escalate risk mitigation and rhythm surveillance
HCM (apical subtype)	Apical aneurysm ± adjacent LGE	High arrhythmic and thromboembolic risk	Consider ICD evaluation and anticoagulation assessment; on follow-up, check aneurysm size/thrombus and new LGE
NDLVC/ALVC	LGE ≥9% of LV mass	Increased risk of major arrhythmic events	Discuss primary-prevention ICD in the context of genotype and clinical factors; track scar burden and RV involvement
NDLVC/DCM	Ring-like circumferential LGE pattern; any LGE with LMNA/FLNC/DSP/RBM20	High arrhythmic substrate/progression risk	Intensify monitoring; consider earlier ICD; avoid high-intensity exercise; act if ring-like LGE appears or expands
DCM	Presence of mid-wall septal LGE (vs. none)	Higher risk of SCD, HF events, and non-response to GDMT alone	Consider device therapy candidacy (ICD/CRT as indicated); track for new or expanding scar
Diffuse fibrosis burden (HCM/DCM/NDLVC)	ECV above site-specific 95th percentile (often ≳27–30% at 1.5T^a^)	Worse outcomes/remodelling risk; treat-to-target marker	Aim for absolute ↓ECV ≥2 percentage points; if ECV rises, reassess therapy intensity/adherence and contributors (BP, ischemia)
AL-CM	Decrease in native T1 ≈≥50 ms from baseline (sequence- and field-specific)	Hematologic/organ response with improved tissue composition	Continue therapy; if T1/ECV rise despite hematologic response, evaluate for cardiac progression and adjust regimen
ATTR-CM	Tafamidis: stable ECV/T1/LGE; RNA-interference: absolute ↓ECV (e.g. ≥2 pp)	Stabilization (tafamidis) or regression (gene silencing)	Maintain therapy when stable; confirm protocol comparability; if ECV/T1 increase, investigate adherence/progression
Fabry disease	Native T1 below site-specific lower limit (storage); emergence of LGE	Low T1 = sphingolipid storage; LGE = advanced/less reversible injury and higher risk	Initiate/continue ERT or chaperone early; success signalled by T1 trending toward normal; LGE expansion → escalate management
Inflammatory cardiomyopathy subset (across phenotypes)	T2 mapping increase beyond site repeatability (≈≥2–3 ms at 1.5T)	Active oedema/inflammation and risk of adverse events while active	Treat the driver (e.g. immunomodulation if indicated); repeat T2 in 6–12 weeks; defer heavy exercise until normalizes

^a^Field- and sequence-dependent reference values: use your lab’s normal ranges and repeatability (RCV). Thresholds above are pragmatic and intended to exceed inter-study variability. Integrate imaging with clinical factors and genotype; do not use a single threshold in isolation for device decisions.

ACM, arrhythmogenic cardiomyopathy; AL-CA, amyloid light-chain cardiomyopathy; ALVC, arrhythmogenic left ventricular cardiomyopathy; ARVC, arrhythmogenic right ventricular cardiomyopathy; ATTR-CA, amyloid transthyretin cardiac cardiomyopathy; BP, blood pressure; CMR, cardiovascular magnetic resonance; DCM, dilated cardiomyopathy; ECV, extracellular volume; GDMT, guideline-directed medical therapy; HCM, hypertrophic cardiomyopathy; HF, heart failure; ICD, implantable cardioverter-defibrillator; LGE, late gadolinium enhancement; LV, left ventricle/left ventricular; NDLVC, non-dilated left ventricular cardiomyopathy; pp, percentage points; RNAi, RNA interference; RV, right ventricle/right ventricular; SCD, sudden cardiac death; T1, longitudinal relaxation time (native T1); T2, transverse relaxation time; VA, ventricular arrhythmias.

## Practical timing of serial CMR in cardiomyopathies

A baseline CMR study is fundamental in the diagnostic work-up of cardiomyopathies because it defines the reference morphologic, functional, and tissue phenotype against which subsequent changes are interpreted. Beyond diagnosis, baseline CMR is particularly valuable before major therapeutic decisions, including ICD or CRT consideration, as biventricular volumes and function, LVEF, RV involvement, and the extent, pattern, and localization of LGE may refine risk stratification, identify persistent arrhythmogenic substrate, and, in CRT candidates, help estimate the likelihood of response and guide lead-target selection away from transmural or extensive scar.^60,61^ In longitudinal follow-up, the timing of repeat CMR should be individualized according to phenotype, baseline severity, treatment milestones, and the likelihood that imaging findings will alter management. Factors favouring earlier reassessment include initiation or escalation of disease-modifying therapy (e.g. CMIs in HCM or ERT in Fabry disease), the need to document reverse remodelling in DCM or NDLVC, new ventricular arrhythmias or syncope, biomarker worsening, suspected inflammatory activity, evolving RV dysfunction, suspected progression of LGE burden or topology, and reassessment before device implantation. Conversely, in clinically stable patients with unchanged phenotype (i.e. symptoms, ECG, Holter monitoring, and echocardiography) and no expected therapeutic consequence, repeat CMR may be deferred and integrated into longer-term surveillance, generally within a pragmatic 2–5-year window, as recommended by the ESC 2023 Guidelines on Cardiomyopathies.^1^

**Figure 4:**
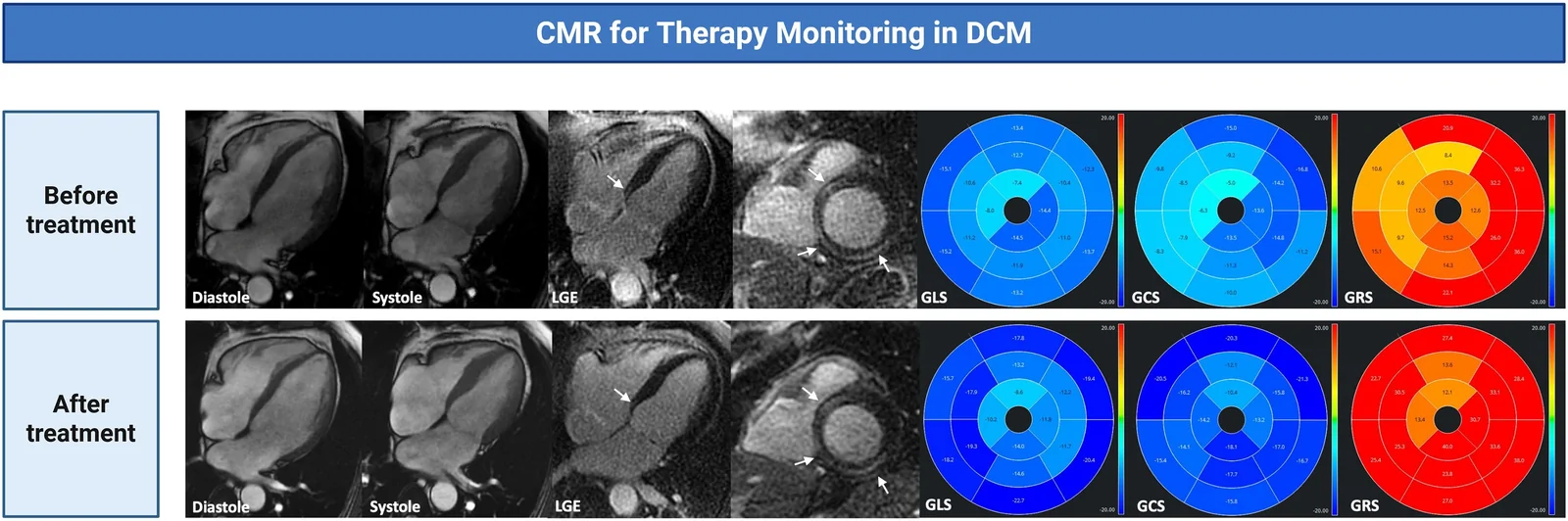
Cardiovascular magnetic resonance (CMR) for therapy monitoring in dilated cardiomyopathy (DCM). Evolution of dilated cardiomyopathy in a patient with pathogenic titin gene mutation. Before therapy (top row), he had 38% left ventricular ejection fraction, midwall late gadolinium enhancement (LGE) in the basal segments of the LV, with a ring-like appearance (white arrows), reduced global strain values: GLS (global longitudinal strain) −12%, GCS (global circumferential strain) −8%, GRS (global radial strain) 10%. After 3 years of optimized medical therapy (bottom row), he presented improved left ventricular ejection fraction (50%), same extent and distribution of LGE (white arrows), improved global strain values: GLS −15%, GCS −13%, GRS 18%.

Special clinical contexts deserve dedicated consideration. In patients presenting with acute myocarditis or myocarditis-like ‘hot-phase’ episodes, an earlier follow-up CMR at approximately 3–4 months may be useful to document oedema resolution, LGE stabilization, and recovery of ventricular function^62^; further imaging can then be reserved for persistent oedema, recurrent injury, or unfavourable clinical evolution. In genotype-positive/phenotype-negative relatives, CMR is mainly used to detect early structural or tissue changes not captured by echocardiography, but the optimal repetition interval remains uncertain and should be individualized according to age, genotype, family history, symptoms, and interval ECG/arrhythmic changes. In the absence of high-risk features or interval clinical changes, a repeat CMR approximately every 5 years may represent a reasonable and pragmatic approach. In DCM and selected NDLVC phenotypes, repeat CMR after treatment optimization may be particularly informative to document reverse remodelling, persistence of scar substrate, and residual arrhythmic risk despite improvement in ejection fraction (*Figure 4*). Once a device has already been implanted, repeat CMR remains feasible in many patients with MRI-conditional systems and appropriate protocols,^23^ but its indication should be more selective and focused on questions likely to change management, such as unexplained clinical deterioration, suspected progression of left- or right-sided disease, reassessment of residual scar burden in arrhythmic syndromes, or evaluation of non-response after CRT. When CMR is limited or non-diagnostic because of device-related artefacts, clinical decision-making should rely on integration with echocardiography, device interrogation, biomarkers, and other imaging modalities.

Overall, robust phenotype-specific evidence for optimal follow-up intervals remains limited, and this should be acknowledged explicitly; current timing is therefore best viewed as a management-oriented framework rather than a fixed schedule. These considerations are summarized in a pragmatic, phenotype-oriented framework in *Table 5*.

**Table 5:** Pragmatic timing of serial CMR for therapy monitoring across cardiomyopathies

Phenotype	Role of baseline CMR	Longer-term surveillance	Triggers for earlier reassessment	Evidence basis
DCM	Defines LV/RV volumes and function, LGE burden/pattern, mapping profile; essential before ICD/CRT to refine risk stratification and guide therapy	If stable, every 2–5 years depending on severity and course	HF worsening, new arrhythmias, biomarker deterioration, pre-ICD/CRT reassessment, suspected inflammation	Guideline-supported for baseline/follow-up; interval largely pragmatic, therapy-driven
NDLVC	Defines fibrosis burden and scar topology; identifies arrhythmogenic substrate	No standardized interval: Every 2–5 years if stable Earlier if evolving phenotype	Syncope, NSVT/VT/VF, ECG modifications, LGE progression, inflammatory activity, genotype-driven reclassification	Limited evidence; interval largely pragmatic, phenotype-driven
ACM	Defines RV/LV involvement, fibro-fatty substrate, and disease phenotype (right-, left-, biventricular)	No standardized interval: Every 2–5 years if stable Earlier if evolving phenotype or after therapeutic/lifestyle changes	Syncope, NSVT/VT/VF, ECG modifications, LGE progression, inflammatory activity, genotype-driven reclassification, progression to LV/biventricular disease, RV dysfunction, ICD decision-making	Limited evidence; interval largely pragmatic
ACM/NDLVC with myocarditis-like ‘hot phase’	Documents oedema, LGE, and ventricular involvement at presentation	3–4 months to assess oedema resolution and LGE stabilization Further imaging only if clinically indicated	Persistent oedema, recurrent symptoms, biomarker elevation, functional deterioration	Limited evidence, based on acute myocarditis data
HCM	Defines wall thickness, LGE burden, apical aneurysm, and risk markers; useful before septal reduction or targeted therapy	Every 3–5 years for SCD risk reassessment and morphologic progression	New symptoms, EF decline, increasing obstruction, arrhythmias, aneurysm suspicion, initiation of CMIs or planning SRT	Supported by guideline statements
Amyloid cardiomyopathy	Defines infiltration burden (LGE, T1, ECV) and baseline for therapy response	Every 2–5 years or individualized based on clinical course	Biomarker worsening, discordant clinical response, suspected progression	Limited evidence, timing largely pragmatic
Storage/metabolic cardiomyopathies (e.g. Fabry, iron overload)	Quantifies LV mass, mapping profile, and fibrosis; baseline before specific therapies	Every 2–5 years depending on phenotype and treatment phase	New symptoms, biomarker changes, emerging LGE, arrhythmias	Limited evidence; therapy- and disease-driven
Genotype-positive/phenotype-negative relatives	Detects early structural/tissue abnormalities not evident on echocardiography	No fixed interval Every ∼5 years in absence of high-risk features or clinical changes	New symptoms, ECG/Holter abnormalities, high-risk genotype, phenotypic transition	Limited evidence; individualized, family/genotype-driven
Patients with implanted ICD/CRT devices	Pre-implant CMR critical for phenotype and scar assessment; post-implant CMR selective	Selective, not routine	Clinical deterioration, recurrent arrhythmias, CRT non-response, suspected progression (MRI-conditional devices)	Feasibility supported; indication is question-driven

Suggested timing of repeat CMR is primarily management-driven and should be individualized according to phenotype, baseline severity, treatment milestones, and likelihood of impacting clinical decisions.

ACM, arrhythmogenic cardiomyopathy; ARVC, arrhythmogenic right ventricular cardiomyopathy; CMR, cardiovascular magnetic resonance; CRT, cardiac resynchronization therapy; DCM, dilated cardiomyopathy; ECG, electrocardiogram; EF, ejection fraction; HCM, hypertrophic cardiomyopathy; HF, heart failure; ICD, implantable cardioverter-defibrillator; LGE, late gadolinium enhancement; LV, left ventricle/left ventricular; NDLVC, non-dilated left ventricular cardiomyopathy; NSVT, non-sustained ventricular tachycardia; RV, right ventricular; SCD, sudden cardiac death; VT, ventricular tachycardia; VF, ventricular fibrillation.

## CMR-based surrogate endpoints

Because CMR maps directly onto core disease processes, it offers biological specificity together with high reproducibility and sensitivity to change over weeks to months. These attributes make it well suited for therapeutic decision-making in phase II trials, dose finding, and as primary or key secondary endpoints in pivotal studies, particularly when hard outcomes would require prohibitive sample sizes or follow-up. Predictive validity reinforces this role: across phenotypes, greater LGE burden, elevated native T1/ECV, adverse strain, and unfavourable remodelling consistently associate with arrhythmias, heart-failure progression, and mortality, lending prognostic weight to CMR-based efficacy signals generated during therapy monitoring.

Endpoint selection aligns with mechanism. For unloading or sarcomere modulation, changes in LVMI, MWT, chamber volumes, and EF (augmented by early shifts in GLS) serve as canonical structural surrogates. In obstructive HCM, CMIs provide the clearest example to date: mavacamten produced CMR-documented reductions in LVMI and MWT with preserved EF, a prototypical remodelling signature that paralleled clinical benefit.^40^ Consistently, in Phase 3 studies, CMR-derived LVMI has been incorporated among secondary endpoints. In MAPLE-HCM,^63^ aficamten was associated with a numerical reduction in LVMI (−6.9 g/m^2^ vs. −3.8 g/m^2^ with metoprolol), although this did not reach statistical significance, whereas in EXPLORER-CN,^64^ mavacamten led to a significant decrease in LVMI compared with placebo (mean difference −30.8 g/m^2^; 95% CI −41.6 to −20.1). These imaging changes paralleled improvements in haemodynamics, functional capacity, and biomarkers. Notably, HCM currently represents the sole cardiomyopathy in which CMR-derived structural parameters have been systematically used as secondary endpoints in randomized trials, whereas evidence in other phenotypes remains limited.

For antifibrotic or disease-modifying strategies in DCM, NDLVC, and the arrhythmogenic spectrum, native T1 and ECV index matrix remodelling, while stability or regression of LGE (when biologically plausible) refines arrhythmic substrate assessment, ideally alongside strain.^16,39^ In amyloid cardiomyopathy, stabilization of ECV/native T1/LGE with TTR stabilization (tafamidis) contrasts with absolute ECV reductions under gene-silencing therapies, aligning with biomarker and functional gains and supporting their use as substrate-level surrogates.^49,50^ When inflammation is the target, T2 mapping provides an objective marker of oedema quiescence; for ischaemia or microvascular interventions, quantitative perfusion captures the intended physiological improvement.

Robust trial design pre-specifies a biologically coherent primary imaging effect, favours absolute change from baseline, and anchors interpretation to a site-validated minimal detectable change, with responder analyses to aid clinical translation. Acquisition and analysis are standardized: fixed scanner and field strength where feasible; locked sequences; consistent contrast dose and post-contrast timing with contemporaneous haematocrit for ECV (or a declared synthetic haematocrit); and blinded core-lab reading with predefined segmentation rules and phantom-based QA to limit drift. Longitudinal statistics account for repeated measures and missing data, with multiplicity control when imaging and clinical endpoints are paired. Confidence strengthens when structural, tissue, and functional domains move concordantly and when imaging changes track with biomarkers such as NT-proBNP in amyloid cardiomyopathy. Safety monitoring can be embedded within the same scans (volumes/EF for negative remodelling and oedema metrics for inflammatory toxicity), particularly relevant for gene or immune-modulating therapies.

Transparent reporting details vendors, field strengths, sequence parameters, software versions, observer variability, and repeatability coefficients for chosen endpoints to support cross-trial comparison and regulatory dialogue. Common pitfalls (measurement drift, vendor effects, physiological confounders, over-interpretation of sub-MDC deltas, and LGE segmentation variability) are mitigated by harmonized protocols, QA, and core-lab adjudication. Taken together, CMR supplies quantitative, biologically meaningful, and reproducible endpoints that can accelerate therapeutic development across cardiomyopathies: detecting signal early, informing dose selection, and, when aligned with improvements in symptoms, function, and events, supporting surrogate claims in registrational pathways.^4^ However, while these biomarkers are well established in phase II studies, their validation as surrogate endpoints for clinical outcomes in phase III trials remains incomplete and requires further prospective confirmation.

CMR biomarkers as surrogate endpoints according to therapeutic mechanism are summarized in *Table 6*.

**Table 6:** Cardiovascular magnetic resonance biomarkers as surrogate endpoints by therapeutic mechanism

Mechanism	Primary imaging endpoint(s)	Typical time-to-signal	Trial role	Standardization notes
Unloading (neurohormonal therapy, afterload reduction, CRT)	↓LVESV (≥10–15%), ↑LVEF (≥5 pp), ↓LVEDV (≥5–10%), ↓LA volume; improved GLS; in RV disease: ↑RVEF/↓RVESV	≈3–6 months for volumes/EF; GLS may improve earlier	Often primary for phase II (go/no-go); key secondary in pivotal HF/device trials	Fixed scanner/field; identical cine stack and segmentation rules; report minimal detectable change; blinded core-lab reading
Sarcomere modulation (myosin inhibitors)	↓LV mass index, ↓max wall thickness, ↓LA volume with preserved EF; supportive improvement in GLS	≈3–7 months (e.g. ∼30 weeks in exemplar studies)	Mechanistic primary in phase II; supportive/secondary in pivotal HCM trials (safety: monitor EF)	Consistent planes for thickness; measure in diastole; avoid partial-volume; report vendor/sequence
Antifibrotic/disease-modifying (matrix)	↓Native T1 (beyond site repeatability, ∼≥20–30 ms at 1.5T), ↓ECV (≥2 pp); stable or ↓LGE (when biologically plausible); improved GLS	Typically ≥6–12 months for mapping; strain may change earlier	Phase II primary (antifibrotic signal); secondary/tertiary in pivotal trials	Lock T1/T2 sequences and field; for ECV keep contrast dose/timing and contemporaneous Hct constant; pre-specify responder thresholds
Anti-inflammatory	↓T2 mapping (beyond site repeatability, ∼≥2–3 ms at 1.5T); resolution of oedema; ±LGE regression in subsets	≈6–12 weeks for oedema signal; LGE change, if any, over months	Phase II mechanistic primary; secondary in disease-activity trials	Use T2-prepared SSFP; replicate slice planes and HR; QC artifacts; avoid sequence swaps across time points
Anti-amyloid (stabilizers and silencers)	Stabilizers (tafamidis): stable ECV/T1/LGE with preserved function; Silencers (RNAi/ASO): absolute ↓ECV with stable or improved function/strain	Stabilization typically evident by 6–12 months; ECV regression with silencers by ∼12 months	Primary/secondary in phase II; key secondary in pivotal ATTR/AL programs; substrate-level surrogate aligned with biomarkers	Strict ECV protocol (contrast dose/timing, contemporaneous Hct); fixed mapping sequence/field; consider multi-organ ECV in AL
Gene therapy (e.g. Fabry, Danon, ATTR editing)	Disease-specific: Fabry → ↑native T1 toward normal and ↓LVMI; ATTR editing → ↓ECV; stabilization or improvement of strain/volumes	Initial signal ≈3–12 months; structural remodelling over 6–24 months	Often primary imaging surrogate in early-phase/rare-disease trials; supportive in later phases	Core-lab with locked protocols; mapping sequence/field fixed; for amyloid include ECV; define organ panel when systemic involvement is expected

Time-to-signal windows are pragmatic and depend on disease severity, loading conditions, and mechanism. Interpret absolute deltas against site-specific repeatability (RCV) and corroborate with clinical and biomarker changes. For all mapping/ECV endpoints, keep acquisition and analysis constants fixed across time.

AL, amyloid light-chain; ASO, antisense oligonucleotide; ATTR, amyloid transthyretin; CRT, cardiac resynchronization therapy; ECV, extracellular volume; EF, ejection fraction; GLS, global longitudinal strain; HCM, hypertrophic cardiomyopathy; Hct, haematocrit; HF, heart failure; LA, left atrium; LGE, late gadolinium enhancement; LV, left ventricle/left ventricular; LVEDV, left ventricular end-diastolic volume; LVEF, left ventricular ejection fraction; LVESV, left ventricular end-systolic volume; LVMI, left ventricular mass index; pp, percentage points; QC, quality control; RCV, repeatability coefficient; RNAi, RNA interference; RV, right ventricle/right ventricular; RVEF, right ventricular ejection fraction; RVESV, right ventricular end-systolic volume; SSFP, steady-state free precession; T1, longitudinal relaxation time; T2, transverse relaxation time.

## Future perspectives

CMR has moved beyond diagnosis to become the engine of longitudinal decision-making across cardiomyopathies. Its core measures (cine remodelling indices, LGE scar architecture, and quantitative mapping) offer complementary views of structure and substrate. In practice, the most actionable signals are those that exceed site-specific minimal detectable change and move concordantly across domains. Interpretation remains phenotype-aware: in dilated and arrhythmogenic disease, substrate biology (LGE topology, T1/ECV trajectories, emerging RV involvement) drives risk protection; in HCM, CMR verifies sarcomere-targeted remodelling; in amyloid, stability vs. regression on mapping/ECV distinguishes mechanisms of action. When standardized, these metrics also function as credible surrogate endpoints for trials in settings where hard outcomes accrue slowly.

The research agenda now centres on making CMR changes actionable, comparable, and trial-ready. Priorities include prospective, multi-centre validation of prespecified ΔT1/ΔECV/Δstrain and scar-topology changes against clinical events with blinded core-lab pipelines; large-scale harmonization efforts—vendor-neutral sequences, travelling-phantom programmes, and shared repeatability registries—to support trust in small longitudinal deltas.

Quantitative perfusion CMR is increasingly recognized as a robust tool for the assessment of coronary microvascular dysfunction across cardiomyopathies. However, while its role in disease characterization and risk stratification is relatively well established, its application in serial therapy monitoring remains less defined and requires further standardization and validation. With continued technical refinement, stress MBF and MPR may evolve into complementary biomarkers for tracking treatment response and improving longitudinal risk assessment.

Artificial intelligence may further expand the role of CMR in therapy monitoring by improving automation, reproducibility, and multimodal integration.^65^ Deep learning tools are increasingly able to automate ventricular segmentation, quantify scar burden, and derive mapping biomarkers such as native T1 and ECV with lower observer variability and greater scalability, potentially making longitudinal assessment more standardized across centres.^66^ Beyond automation, radiomics and machine learning may extract clinically relevant information from cine texture, LGE architecture, and pixel-wise mapping data that is not captured by conventional summary metrics alone. A particularly promising direction is the integration of CMR features with clinical, ECG, and genetic data to generate composite models for diagnosis, risk stratification, and prediction of therapeutic response.^67^ In selected settings, such approaches may also support contrast-sparing strategies, for example through virtual native enhancement or AI-assisted fibrosis assessment.^68^ Looking further ahead, digital twin and *in silico* trial frameworks may combine imaging, genotype, and longitudinal clinical data to simulate disease trajectories and treatment effects at the individual level, although these applications still require substantial validation before routine clinical implementation.

Additional targets include integrating CMR scar topology with electroanatomic and device data to refine ICD selection and ablation strategies in left-dominant ACM; testing quantitative perfusion as a mechanism-linked endpoint for microvascular dysfunction; and building therapy-specific monitoring frameworks for gene/RNA approaches and systemic AL (including multi-organ ECV panels). Emerging tissue-characterization approaches, including diffusion tensor imaging and T1ρ mapping,^69,70^ may provide complementary insight into myocardial microstructure and fibrosis activity, but their current role in serial therapy monitoring remains exploratory and requires further technical and clinical validation. Finally, pragmatic implementation studies (treat-to-target strategies, CMR-first device pathways, cost-effectiveness analyses, and streamlined protocols for paediatrics, MR-conditional devices, and limited breath-hold capacity) will determine real-world impact.

Together, these directions position CMR not merely as a sensitive measuring tool but as a standardized, regulation-ready platform that informs therapy intensity, rhythm protection, and device timing while accelerating development of disease-modifying treatments.

## Data Availability

No new data were generated or analysed in support of this research.

## Lead author biography

Dr. Alberto Aimo, MD, PhD, FESC, FHFA, is a cardiologist at the Fondazione Toscana Gabriele Monasterio, a division of the Italian National Research Council, and a researcher at the Scuola Superiore Sant’Anna in Pisa, Italy. His research primarily focuses on heart failure and cardiomyopathies, with an emphasis on the use of biomarkers and advanced imaging for diagnosis and risk stratification, as well as drug and device therapies and structural interventions in heart failure.
